# Retraction Note: Upregulation of ARHGAP30 attenuates pancreatic cancer progression by inactivating the β-catenin pathway

**DOI:** 10.1186/s12935-025-03710-4

**Published:** 2025-02-27

**Authors:** Yongping Zhou, Zhiyuan Hua, Ye Zhu, Liying Wang, Fangming Chen, Ting Shan, Yunhai Zhou, Tu Dai

**Affiliations:** 1 Department of Hepatobiliary Surgery, Wuxi Second Hospital, Nanjing Medical University, No. 68 Zhongshan Road, Wuxi, 214000 People’s Republic of China; 2 Department of Imaging, Wuxi Second Hospital, Nanjing Medical University, Wuxi, 214000 People’s Republic of China; 3 Department of General Surgery, Wuxi Second Hospital, Nanjing Medical University, No. 68 Zhongshan Road, Wuxi, 214000 People’s Republic of China

**Cancer Cell International (2020) 20:225**

10.1186/s12935-020-01288-7

The Editor-in-Chief has retracted this article. After publication, concerns were raised regarding some of the figures, specifically:

- Fig. 1d ARHGAP30, Fig. 2d ARHGAP30 and Fig. 4e b-catenin blots appear to share some of the same bands arranged in different order and orientation.
- The GAPDH western blots in Figures 4e and 5c appear highly similar, and also appear to have been used in another article from a different group that was submitted and published within a similar time frame [1] (now retracted).

The authors have been unable to provide the underlying raw data upon request. The Editor-in-Chief therefore no longer has confidence in the presented data.

Tu Dai has not explicitly stated whether they agree with this retraction. None of the other authors have responded to any correspondence from the editor or publisher about this retraction.

